# A rare cause of unilateral leg swelling: May–Thurner Syndrome

**DOI:** 10.1093/jscr/rjad232

**Published:** 2023-05-03

**Authors:** Hamid Channane, Panagiotis M Spiliotis, Andreea M Sandica, Iurii Snopok, Richard Viebahn

**Affiliations:** Department of Surgery, Ruhr-University Bochum, Knappschaftskrankenhaus, Bochum 44892, Germany; Department of Surgery, Ruhr-University Bochum, Knappschaftskrankenhaus, Bochum 44892, Germany; Department of Surgery, Ruhr-University Bochum, Knappschaftskrankenhaus, Bochum 44892, Germany; Department of Surgery, Ruhr-University Bochum, Knappschaftskrankenhaus, Bochum 44892, Germany; Department of Surgery, Ruhr-University Bochum, Knappschaftskrankenhaus, Bochum 44892, Germany

## Abstract

Common left iliac vein compression, otherwise known as May–Thurner Syndrome (MTS), is a medical condition that refers to chronic compression of an anatomical variant of the left iliac vein by the overlying right common iliac artery and is a predisposing factor for deep vein thrombosis of the left lower limb (LDVT). Although MTS is not often, its true prevalence is underestimated due to misdiagnose, fact that can result to life-threatening conditions such as the development of LDVT and pulmonary embolism. In this paper, we present a case of MTS presenting at our department with unilateral leg swelling without LDTV that was treated through endovascular management along with long-term anticoagulation. With this presentation, the authors wish to emphasise the importance of MTS as a frequently under-diagnosed condition that needs to be ruled out in unilateral left leg swelling with or without LDVT.

## INTRODUCTION

May–Thurner Syndrome (MTS), otherwise known as iliac vein compression syndrome or Crockett-Syndrome refers to the compression of the left common iliac vein by the right common iliac artery. Due to an anatomic variation, the right common iliac artery over-crosses the left common iliac vein pressing it against the lumbar spine. The compression impairs the venal blood flow by slowing it down, possibly causing thrombotic formations [1]. This phenomenon has been described first by May and Thurner in 1957 [2]. Still in many cases, the iliac vein compression syndrome is not properly diagnosed. Based on the data published until now, it seems to be more common in the female than in the male population [3, 4]. The compression of the iliac vein as described above can remain asymptomatic or associated with deep vein thrombosis (DVT) and/or edema of the left lower extremity. If untreated and associated with deep vein thrombosis, MTS can be complicated by pulmonary embolism (PE), venous rupture, retroperitoneal haematoma, rhabdomyolysis or TVT resistant to therapy [5, 6, 7]. A digital substraction venography (DSV) as well as the CT-venography (CTV) are standard diagnostic measures for MTS [8, 9]. The first diagnosed cases of MTS have been treated with anticoagulation therapy alone. This stopped the extension of the thrombosis but did not solve the mechanic compression causing the thrombus to form, thus was not able to prevent complications as listed above. Our case stresses the importance of MTS as a differential diagnosis in swelling of the left lower limb to choose the proper course of treatment, including endovascular procedures along with anticoagulation therapy [10, 11].

## CASE

**Figure 1:**
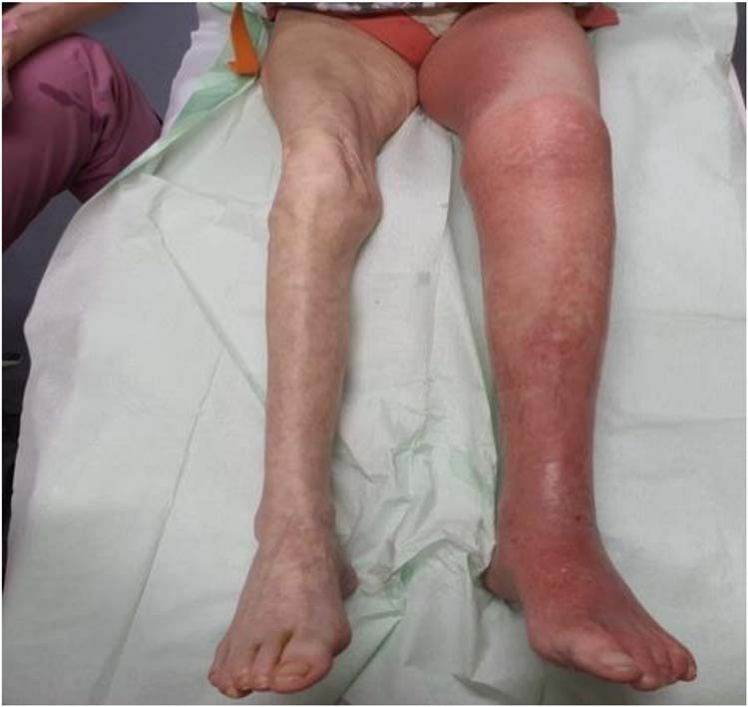
Clinical findings upon admission. Swelling of the left lower limb.

**Figure 2:**
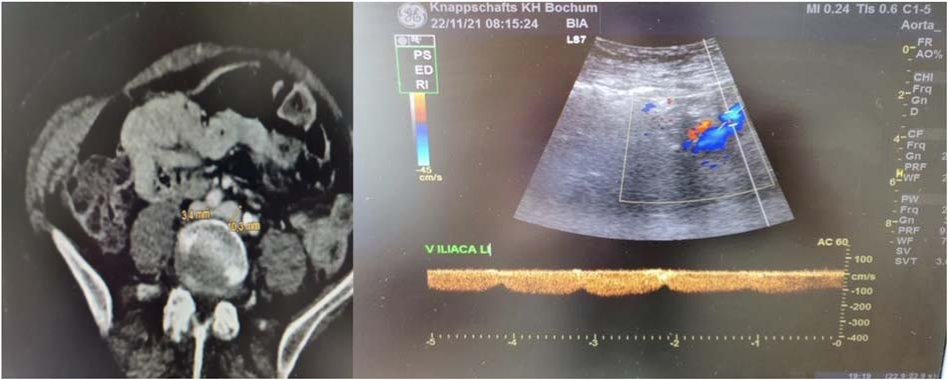
Radiological findings. Thrombosis of the left common/extern iliac vein as seen upon CT-scan and sonography.

We present the case of a 61-year-old woman who was diagnosed with swelling of the left lower limb for 2 weeks (Fig. 1). She had already been admitted to another hospital and dismissed from the department for vascular surgery after a DVT had been ruled out. Patient history included smoking and regular consumption of alcohol. Trauma, tumor disease, major surgery within 6 weeks before symptom start and prior DVT were ruled out. Patient vitals check showed no abnormalities. When the patient was presented to our department, a DVT was again ruled out by sonography. A CT-angiography and further sonogram of the iliac vessels showed a relevant stenosis of the left common/extern iliac vein (Fig. 2).

**Figure 3:**
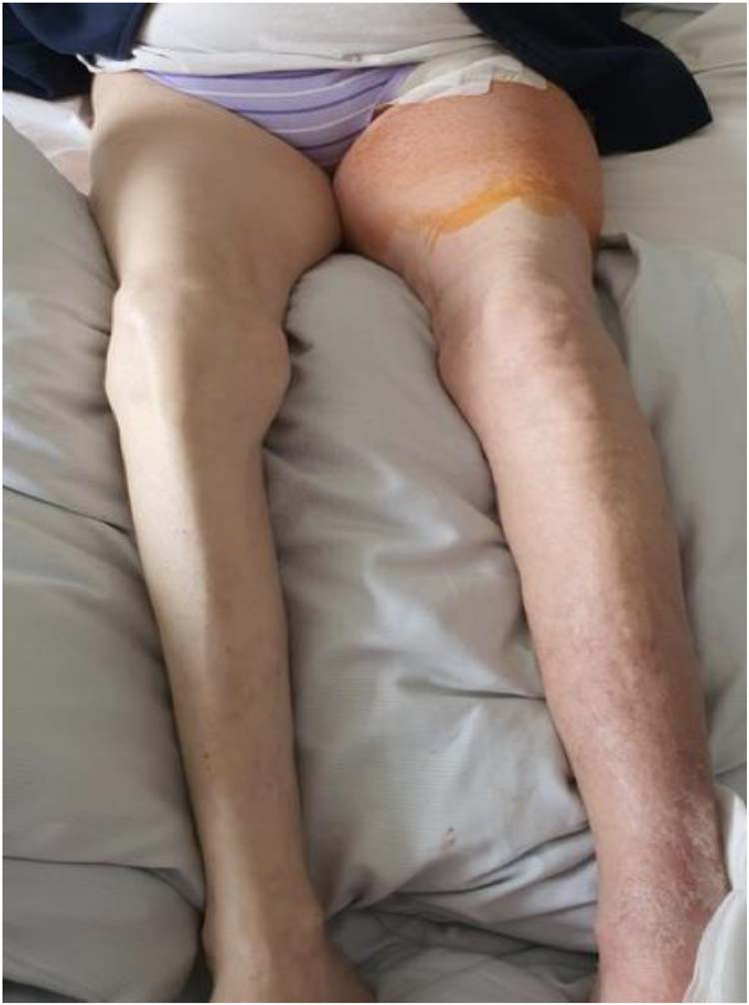
Clinical findings upon dismission. Notable improvement in swelling of the lower left limb.

For the possible diagnosis of MTS based on the radiological findings, the patient underwent venotomy with clot debulking, phlebography and angioplasty of the left common and extern iliac vein. Before intervention, she was put on heparin for therapeutic anticoagulation. After the intervention, swelling of the left lower limb improved significantly (Fig. 3). The patient was mobilised effortlessly and could be discharged two days after the procedure. On discharge, she showed good perfusion of the left lower limb. Oral anticoagulation was continued after discharge.

## DISCUSSION

MTS describes compression of the left common iliac vein by the right common iliac artery, which can lead to venous thrombosis. It is an important differential diagnosis in patients with DVT of the left lower limb, especially in patients without risk factors for thrombosis. Even in atypical presentations with swelling of the left lower limb without DVT like in our case, MTS should be ruled out to prevent further complications like therapy-resistant DVTs and PE. As therapeutic anticoagulation, as a monotherapy, is not able to prevent complications, treatment for MTS should include endovascular thrombolysis and release of the compression on the common iliac vein along with anticoagulation therapy. When comparing endovascular treatment methods, balloon angioplasty alone had been showed to be less effective upon long-term than venous stenting. However, venous stenting is challenging because of the risk of stent deformation due to arterial pulsation and difficulties in choosing the proper landing zones [12]. Implant of multiple stents should be avoided to minimise the risk for in-stent thrombosis. Developments in iliac venous stents have been leading to a 6-year primary patency rate up to 79% [12–15]. Intravascular ultrasound can help to identify the proper landing zones [12].

Compared with endovascular treatment methods, venotomy and clot de-bulking is rarely performed for MTS, also due to improvement in thrombolysis techniques [12–15]. We performed this in our patients along with PTA of the common iliac vein with good clinical outcome upon a long-term follow up. Our findings confirm that venotomy is still a viable course of treatment for MTS, especially if thrombolysis is contraindicated.
